# Zinc Oxide Synthesis from Extreme Ratios of Zinc Acetate and Zinc Nitrate: Synergistic Morphology

**DOI:** 10.3390/ma15020570

**Published:** 2022-01-13

**Authors:** Sujittra Kaenphakdee, Pimpaka Putthithanas, Supan Yodyingyong, Jeerapond Leelawattanachai, Wannapong Triampo, Noppakun Sanpo, Jaturong Jitputti, Darapond Triampo

**Affiliations:** 1 Department of Chemistry and Center of Excellence for Innovation in Chemistry, Faculty of Science, Mahidol University, Phuttamonthon Sai 4 Road, Salaya, Nakhon Pathom 73170, Thailand; sujittra.kae@student.mahidol.ac.th (S.K.); pimpaka.put@student.mahidol.ac.th (P.P.); 2 Institute for Innovative Learning, Mahidol University, Phuttamonthon Sai 4 Road, Salaya, Nakhon Pathom 73170, Thailand; supan.yod@mahidol.edu; 3 National Nanotechnology Center (NANOTEC), National Science and Technology Development Agency (NSTDA), Klong Luang, Pathum Thani 12120, Thailand; jeerapond@nanotec.or.th; 4 Department of Physics, Faculty of Science, Mahidol University, Phuttamonthon Sai 4 Road, Salaya, Nakhon Pathom 73170, Thailand; wannapong.tri@mahidol.edu; 5 SCG Chemical Co., Ltd., Siam Cement Group (SCG), 271 Sukhumvit Road, Muang District, Rayong 21150, Thailand; noppsanp@scg.com (N.S.); jaturojii@scg.com (J.J.)

**Keywords:** NIR-shielding, ZnO, thermal insulation, coating pigment

## Abstract

The synthesis of ZnO comprising different ratios of zinc acetate (ZA) and zinc nitrate (ZN) from the respective zinc precursor solutions was successfully completed via a simple precipitation method. Zinc oxide powders with different mole ratios of ZA/ZN were produced—80/1, 40/1, and 20/1. The crystallinity, microstructure, and optical properties of all produced ZnO powders were characterized using X-ray diffraction (XRD), scanning electron microscopy (SEM), and UV-Vis-NIR spectrophotometry. The average agglomerated particle sizes of ZnO-80/1, ZnO-40/1, and ZnO-20/1 were measured at 655, 640, and 620 nm, respectively, using dynamic light scattering (DLS). The optical properties of ZnO were significantly affected by the extreme ratio differences in the zinc precursors. ZnO-80/1 was found to have a unique coral-sheet structure morphology, which resulted in its superior ability to reflect near-infrared (NIR) radiation compared to ZnO-40/1 and ZnO-20/1. The NIR-shielding performances of ZnO were assessed using a thermal insulation test, where coating with ZnO-80/1 could lower the inner temperature by 5.2 °C compared with the neat glass substrate. Due to the synergistic effects on morphology, ZnO-80/1 exhibited the property of enhanced NIR shielding in curtailing the internal building temperature, which allows for its utilization as an NIR-reflective pigment coating in the construction of building envelopes.

## 1. Introduction

The residential component of electricity usage in Thailand comprised approximately 27% of the total electricity consumed in 2020 [1]. The use of thermal insulation materials is one of the most effective procedures for reducing the electricity consumption of buildings and thus decreasing household expenditure. Thermal insulation materials have been used in the envelopes of buildings, such as walls, roofs, and windows [2,3,4,5,6]. In modern buildings, roof and window glass are used for aesthetics and to increase internal brightness, making it is necessary to find a material to coat the glass in order to reduce the internal heat of buildings.

Solar power distribution consists of ultraviolet (UV, 5%), visible (Vis, 43%), and near-infrared (NIR, 52%) energy [7]. More than half of the solar power energy is distributed in the NIR region, which can radiate heat to the earth. In buildings, window glass lacks heat-shielding properties, which leads to the cumulation of internal heat that causes discomfort for occupants. An effective approach to optimizing NIR protection can be achieved by coating the building glass with NIR-reflective pigment. These pigments are widely used in roofs and windows to reduce the inner temperature of buildings due to their NIR radiation properties, which contribute to minimizing the indoor heat build-up. The NIR-reflective pigments can be classified into inorganic and organic compounds [8]. The inorganic NIR-reflective compounds are primarily semiconducting metal oxides, such as antimony tin oxide (ATO) [9,10], indium tin oxide (ITO) [11,12], titanium dioxide (TiO_2_) [13,14], and zinc oxide (ZnO) [15,16]. Among the various kinds of NIR-reflective pigments, ZnO is well known as a multifunctional material due to its unique physical and chemical properties, such as wide bandgap (3.4 eV) [17], high chemical stability [18], and high photostability [19]. It is suitable for use as a coating powder due to its numerous advantages, such as non-toxicity to humans [20,21], biocompatibility [22], low cost [23], and high UV absorption [24,25] and NIR reflection [26] properties. The different conditions used for ZnO synthesis results in different ZnO morphologies. This leads to different NIR-shielding properties [27] as the NIR-shielding performance of ZnO is dependent on its morphology. The synthesis process has a crucial effect on the formation of ZnO morphology. There are various methods for synthesizing ZnO powder, such as hydrothermal, microwave heating, and precipitation techniques. The hydrothermal process is a common procedure involving high temperatures and is used for preparing high-quality nanoscale materials under mild conditions as a calcination process [28]. However, without the calcination process, the resulting ZnO has low electrical conductivity, which results in low thermal conductivity [29], and this contributes to a decrease in the NIR-shielding properties [13]. As for the microwave heating technique, the feedstock was heated quickly and uniformly due to the direct molecular heating caused by the absorbed microwave radiation of polar solvent. Compared to conventional heating (hydrothermal), synthesis techniques involving microwave heating have various advantages, such as in terms of product purity, short synthesis duration, and being environmentally friendly [30]. However, the microwave heating technique requires complex and expensive equipment and could therefore be difficult to use in scale-up manufacturing [31]. In the precipitation method, various kinds of alkalis and zinc sources are employed [32]. The synthesis process begins with a reaction between zinc and hydroxide ions, followed by an aggregation process. Then, the precipitate is collected by filtration or centrifugation. The process of fabricating ZnO powder is simple as it can be easily prepared using simple equipment and is suitable for up-scale, which can facilitate low-cost production. In addition to the synthesis method, several factors in the synthesis process can lead to diverse ZnO morphologies, such as the specific Zn precursor [20,33], pH [34,35], capping agent [26,36,37], reaction time [38,39], and calcination temperature [40,41] that are used.

The synthesis of ZnO from zinc acetate precursor results in a spherical shape [42], while the ZnO prepared from zinc nitrate yields a rod shape [43]. In terms of NIR-shielding performance, the spherical ZnO synthesized by hydrothermal synthesis acts as an excellent barrier to energy in the NIR region, while the rod morphology shows less visible reflectance [44]. In glass coating applications, the NIR-reflective pigment must not only have high NIR reflection but also low visible reflectance to allow visible light to pass through into the buildings. Therefore, the combination of zinc acetate and zinc nitrate may provide a suitable material for NIR shielding.

In this work, ZnO powders were synthesized using source material containing various mole ratios of zinc acetate and zinc nitrate via a simple precipitation method. The structures, morphologies, and NIR reflection properties of all synthesized ZnO powders were investigated using X-ray diffraction (XRD Bruker AXS, Karlsruhe, Germany), scanning electron microscopy (SEM SU-3500 Hitachi, Tokyo, Japan), and UV-Vis-NIR spectrophotometry (Agilent Technologies, Santa Clara, CA, USA). The ZnO powders were coated onto a glass substrate to assess their thermal insulation performance. Moreover, the mentioned properties of ZnO obtained from pure zinc acetate and zinc nitrate were also systematically studied.

## 2. Materials and Methods

### 2.1. Materials

Zinc nitrate hexahydrate (Zn(NO_3_)_2_·6H_2_O, Loba Chemie, Mumbai, India 98%) and zinc acetate dihydrate (Zn(CH_3_COO)_2_·2H_2_O, Daejung Chemicals, Gyeonggi-do, Korea 98%) were chosen as zinc precursors of ZnO powders. Hexamethylenetetramine ((CH_2_)_6_N_4_, Ajax Finechem, New South Wales, Australia), monoethanolamine (HOC_2_H_4_NH_2,_ Sigma Aldrich, St. Louis, MO, USA, 99%), and 2-methoxyethanol (HOC_2_H_4_OCH_3_, Alfa Aesar, Ward Hill, MA, USA 99%) were used as received without further purification. Microscope slides were used as substrates representing window glass.

### 2.2. Synthesis of ZnO with Different Ratios of ZA/ZN

**Figure 1:**
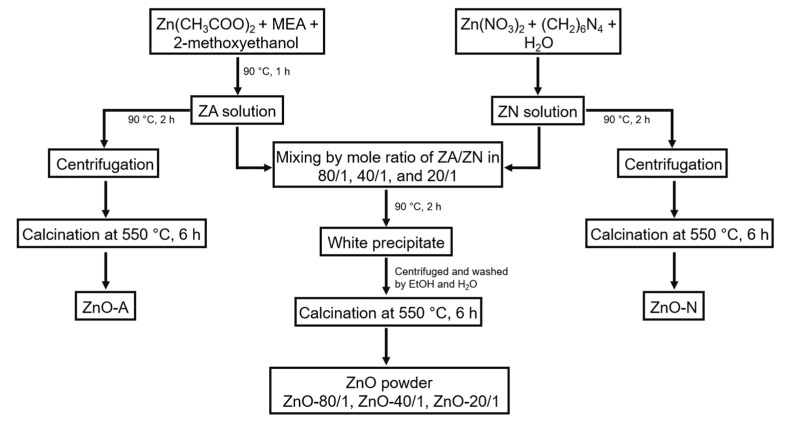
Schematic flow chart of ZnO powder preparation yielding ZnO-80/1, ZnO-40/1, ZnO-20/1, ZnO-A, and ZnO-N.

The synthesis of ZnO particles with different molar ratios of zinc acetate (ZA) and zinc nitrate (ZN) was performed using a simple precipitation method [45]. The synthesis flow chart in Figure 1 summarizes the method used to produce ZnO powders. Typically, the synthesis of ZnO particles began with the preparation of ZA and ZN precursor solutions. The ZA solution was prepared by dissolving 0.6 M ZA and 0.4 M monoethanolamine in 2-methoxyethanol at room temperature with continuous stirring for 30 min before heating at 90 °C for 1 h. The ZN solution was produced by dissolving 0.015 M ZN and 0.015 M hexamethylenetetramine in deionized (DI) water. The ZN solution was subsequently stirred until the solution became clear. Then, ZA and ZN solutions were mixed with ZA/ZN at mole ratios of 80/1, 40/1, and 20/1. During the mixing procedure, the precipitates formed instantly in the mixed solutions. The mixed solutions were subsequently incubated in a temperature-controlled oven at 90 °C for 2 h. The precipitates were washed through several rounds of rinsing with ethanol and then DI water and with centrifugation, then dried in the oven for 12 h. Finally, the samples were calcined at 550 °C for 6 h in a muffle furnace (CWF 1200, Carbolite Gero, Neuhausen, Germany). Calcination is required for the oxidative transformation of Zn${\left(\mathrm{OH}\right)}_{4}^{2-}$ into ZnO [46]. The ZnO particles prepared with the various mole ratios of ZA/ZN at 80/1, 40/1, and 20/1 are referred to as ZnO-80/1, ZnO-40/1, and ZnO-20/1, respectively.

In the preparation of ZnO-A and ZnO-N, instead of mixing the two precursor solutions of various extreme ratios, ZA and ZN solutions were independently heated at 90 °C for 2 h, and the precipitates were collected by centrifugation. The precipitates were calcined at 550 °C for 2 h to obtain the ZnO powder from ZA and ZN solutions, yielding ZnO-A and ZnO-N, respectively.

### 2.3. Sample Characterizations

Data for the analysis of ZnO crystal structures were obtained by a D2 phaser benchtop X-ray diffractometer (Bruker AXS, Karlsruhe, Germany) with Cu–Kα radiation (λ = 1.5406 Å and 2θ = 20–80°). The interval between planes of ZnO powders was calculated using Bragg’s equation [34] as follows:

nλ = 2dsinθ(1)

where n is an integer; λ is the wavelength of the X-ray, which is 1.5406 Å; d is the distance between (hkl) planes, also called the d-spacing; and θ is the angle of incidence of X-rays to the planes. The average crystallite size of ZnO was calculated according to the Debye−Scherrer equation [17], represented in the following formula:

(2) $$D\;=\frac{0.9\lambda }{\beta \cos\theta }$$

where D is the crystallite size, λ is the wavelength of the X-ray, β is the full width at half-maximum (FWHM) of the X-ray diffraction peak in radians, and θ is Bragg’s diffraction angle. The microstructures of all samples were collected by scanning electron microscopy ((SEM) using an SU-3500 (Hitachi, Tokyo, Japan)) with a voltage of 10 kV. The agglomerated particle size analysis of all ZnO powders was performed by dynamic light scattering (DLS) using the Zetasizer nano ZS (Malvern Instruments, Malvern, United Kingdom). For the DLS measurements, the samples were prepared using an ultrasonic dispersion of 0.01 g ZnO in 100 mL ethanol at 25 °C. The efficiency of the NIR shielding of ZnO powders was analyzed using an Agilent Cary UV-Vis-NIR spectrophotometer in the wavelength range of 200–2500 nm.

### 2.4. Thermal Insulation Test

**Figure 2:**
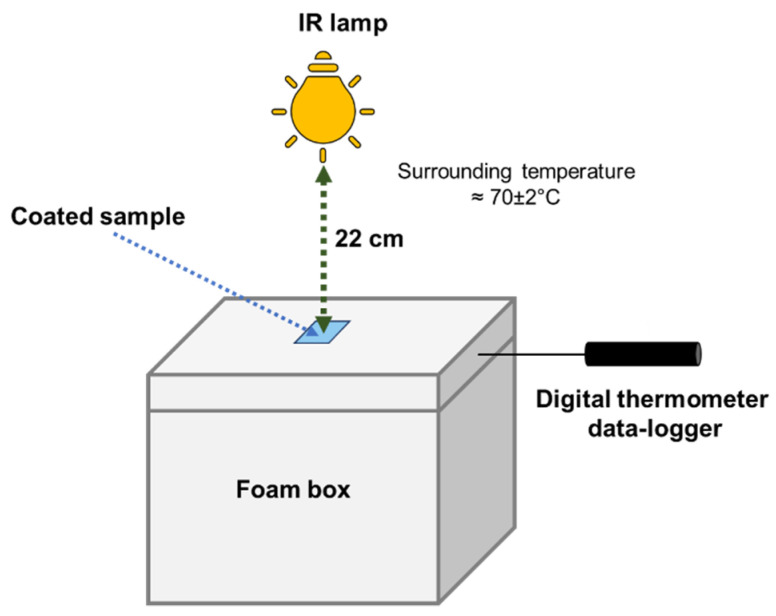
Schematic of the thermal insulation testing setup.

The NIR-shielding performance of actual ZnO powder was investigated by dispersing 10 mg of powder in ethanol then coating it onto the cleaned glass substrates with sizes of 2.5 × 3.8 cm^2^. Then, the coated samples were dried to evaporate the solvent in the oven. The experimental setup for thermal insulation testing is shown in Figure 2. The setup consisted of a polystyrene (PS) foam box with a size of 21 × 30 × 25 cm^3^, representing the house; a 150 W IR lamp, representing the sun (or the heat source); and digital thermometer data-loggers (173T3, Testo, West Chester, United States), which were used to measure the inner temperature of the house. The powder-coated sample was placed on top of the PS box, which mimicked a window of the building. The data-loggers recorded the change in temperature inside the box for 1 h.

## 3. Results and Discussion

### 3.1. SEM

**Figure 3:**
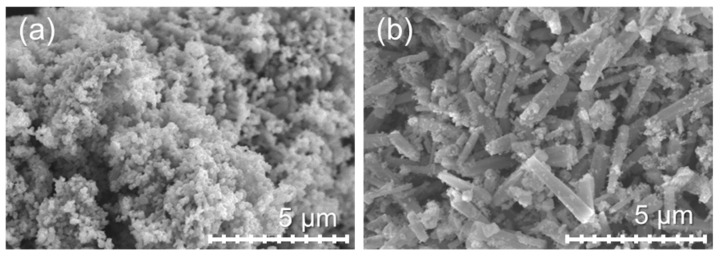
SEM micrographs of ZnO prepared from ZA and ZN precursors: (**a**) ZnO-A and (**b**) ZnO-N.

The morphologies and microstructures of all ZnO powders were investigated using scanning electron microscopy (SEM). Figure 3a depicts the morphology of ZnO powder prepared from the ZA solution. The ZnO-A particles had an average size in the range of 400–500 nm. The ZnO particles show extensive nano- to micron-size aggregation. The cause of the massive aggregation of the ZnO particles can be explained by the particle requirements to reduce the surface energy and increase stability via a thermodynamic-driven process, which results in extensive aggregate formation. In addition, the particles have a large surface area, which increases the collision frequency, causing further aggregation of ZnO particles [47]. The particles of the ZnO-N powder have a submicrorod shape [44] with a size of approximately ~3 μm in length and ~500 nm in diameter, as shown in Figure 3b.

**Figure 4:**
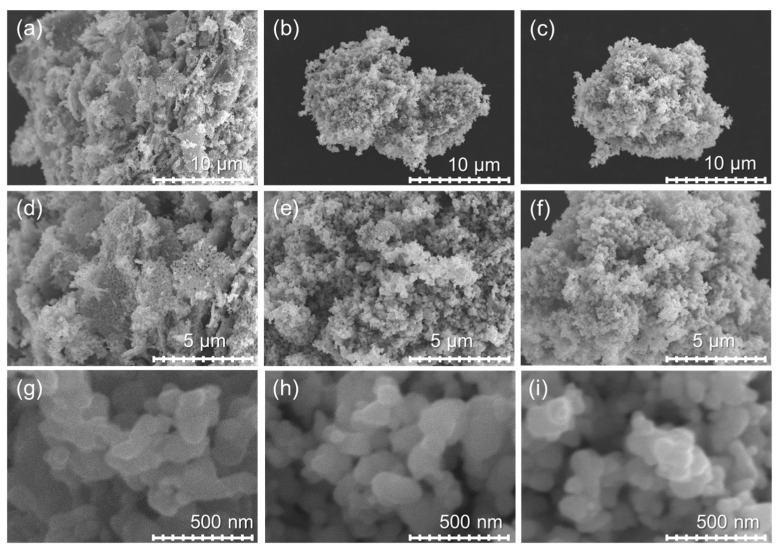
SEM micrographs of ZnO with the different ratios of ZA/ZN captured at 5000×, 10,000×, and 100,000× magnification of (**a**,**d**,**g**) ZnO-80/1, (**b**,**e**,**h**) ZnO-40/1, and (**c**,**f**,**i**) ZnO-20/1, respectively.

ZnO-80/1, ZnO-40/1, and ZnO-20/1 exhibit completely different morphologies and optical properties from those of ZnO-A particles and ZnO-N submicrorods. The agglomerates of ZnO-80/1, ZnO-40/1, and ZnO-20/1 particles in ethanol suspension were sized at 655, 640, and 620 nm, respectively, as measured by DLS. Micrographs of ZnO prepared using these ZA and ZN solutions of extreme ratios captured at 5000×, 10,000×, and 100,000× magnifications are shown in Figure 4. ZnO-80/1 (Figure 4a,d,g) exhibited particles that were attached to other particles to form a coral-sheet structure morphology. The coral-sheet structure was agglomerated, leading to a dense and compact non-uniform stacked sheet ZnO structure. On the other hand, ZnO-40/1 and ZnO-20/1 comprised only particles that were similar to those of ZnO-A. These results show that the morphology and microstructure of ZnO powders were affected by the proportions of ZA and ZN precursors used for synthesis.

### 3.2. XRD

**Figure 5:**
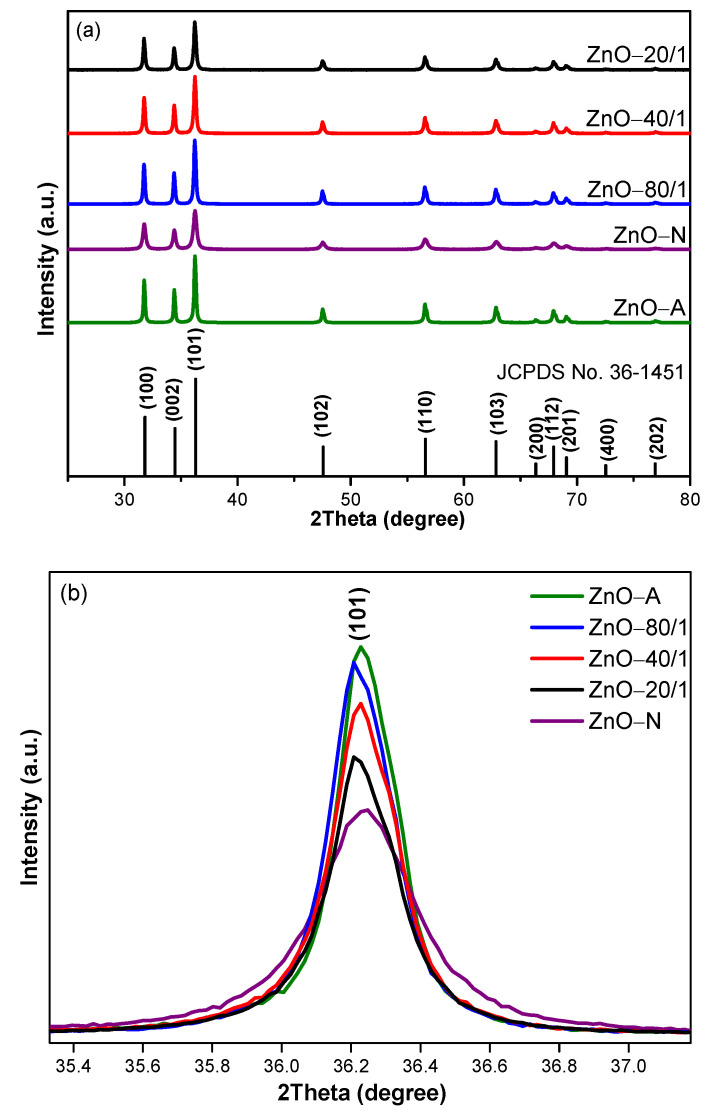
The XRD patterns of (**a**) a wide range of ZnO with different ratios of ZA/ZN and (**b**) the enlarged (101) peak of ZnO-A, ZnO-N, ZnO-80/1, ZnO-40/1, and ZnO-20/1.

The X-ray diffraction patterns of ZnO of the five samples are shown in Figure 5a. Typical planes of polycrystalline ZnO consisting of (100), (002), (101), (102), (110), (103), and (112) were observed. All peaks of ZnO samples were sharp and high in intensity, which indicates a high degree of crystallinity corresponding to polycrystalline ZnO (JCPDS card No. 36-1451). The XRD patterns did not show impurity diffraction peaks, such as of Zn(OH)_2_, which were found in the work of Zhu [48]. The amplified (101) planes of all ZnO samples are shown in Figure 5b. ZnO-A had peaks of the highest intensity, indicating the highest crystallinity, whereas ZnO-N had the lowest crystallinity. As a result, ZnO-80/1, synthesized with the highest proportion of ZA, yielded the highest peak intensity, followed by ZnO-40/1 and then ZnO-20/1. The intensity of the diffraction peaks of ZnO-80/1, ZnO-40/1, and ZnO-20/1 are noticeable, and gradually and proportionally decreased according to ZA. In addition, the ZnO particle samples that increased in all ratios of ZA/ZN solutions had the same hexagonal wurtzite structure. As the amount of ZA solution increased, the peak broadening of all diffraction peaks decreased and the diffraction peak sharpness increased, which indicates a reduction in the lattice strain and an increase in the crystallinity of all mixed ZnO samples, respectively [49]. The peak broadening of the XRD patterns was mainly due to the presence of small nanocrystals in the samples [50]. The peak broadening decreased due to the increase in the proportion of ZA solution, which led to a decrease in the crystallite sizes. The crystallite sizes of all ZnO samples were calculated using the Debye−Scherrer Equation (2). The structure parameter and crystallite size were calculated from the (101) plane, which was the highest intensity plane, as shown in Table 1. The intensity of the diffraction peaks varied significantly from samples prepared using different ZA/ZN solution ratios, indicating that ZA/ZN solution ratios play a vital role in the growth of ZnO crystallites.

### 3.3. UV-Vis-NIR Spectrophotometer

**Figure 6:**
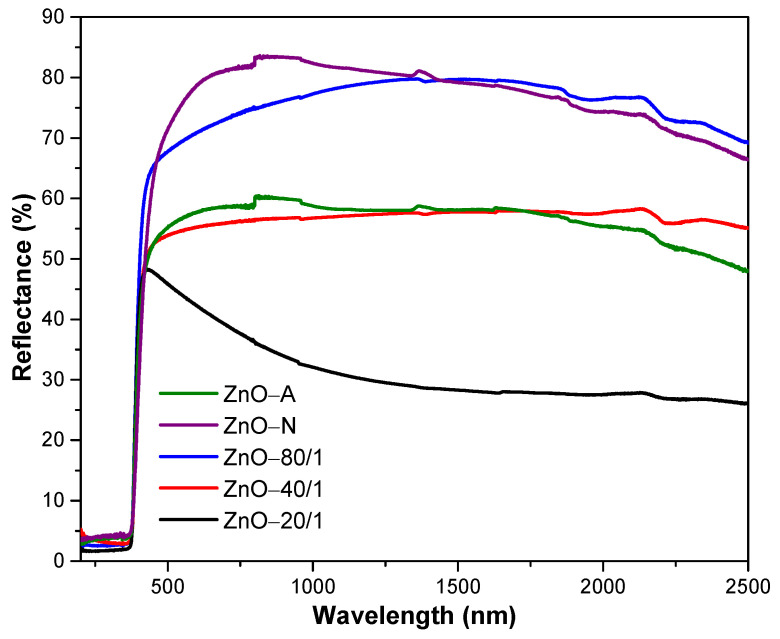
UV-Vis-NIR reflectance spectra of ZnO with different ratios of ZA/ZN precursors.

The UV-Vis-NIR reflectance spectra of ZnO with various ZA/ZN precursor ratios are shown in Figure 6. From the graphs, it can be seen that all samples of ZnO barely reflected in the UV range due to the ZnO powders have excellent absorption in the UV region [51,52]. It is well known that the NIR reflectance property strongly depends on the color, refractive index, and crystalline qualities of inorganic pigment [13]. It can be seen from the graph that all the ZnO samples had high reflectance in the visible region, indicating pigments have a high level of whiteness. The ZnO-N powder presented the highest reflection in the visible range, with 77%, while that of the ZnO-A particle was only 57%. As a comparison between the ZnO-A particle and ZnO-N submicrorod powders, the ZnO-N not only showed a larger particle size but also showed a larger active surface for interacting with the light than ZnO-A. Thus, the reflectance of ZnO-N was significantly higher than that of ZnO-A.

**Figure 7:**
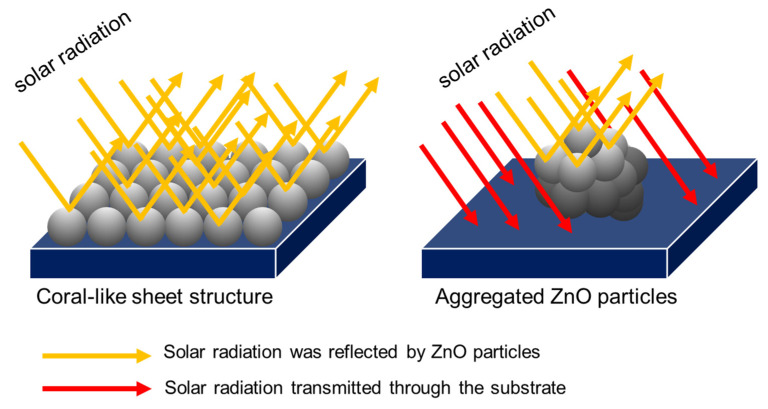
Illustration of solar reflectance at the active site of the coral-sheet structure of ZnO-80/1 versus the aggregated structure of ZnO-40/1 and ZnO-20/1.

The reflection property of mixing ZnO was dramatically affected by the ratio of the ZA/ZN solution. ZnO-80/1, ZnO-40/1, and ZnO-20/1 showed reflectance in the visible range of 71%, 55%, and 42%, respectively. The ZnO-80/1 powder with the highest reflectance resulted from its distinguished morphology. The ZnO-80/1 powder had a coral-sheet structure, which increased the reflection in the visible range by more than that of ZnO-40/1 and ZnO-20/1, which had an aggregated particle structure. Considering the results from SEM and UV-Vis-NIR together, as well as the mixing of various ratios of ZA and ZN solutions, we proposed a model for the high reflectance of ZnO-80/1 with a coral-sheet structure, as shown in Figure 7. The coral-sheet structure is represented by 20 balls tied together to form a sheet. Compared with the same number of aggregated balls, the active site area for reflecting solar radiation was higher in the coral-sheet structure than in the aggregated structure. Thus, ZnO-80/1, with its coral-sheet structure, demonstrated higher solar reflectance than ZnO-40/1 and ZnO-20/1.

The NIR reflectivity range was also influenced by the degree of ZnO particle aggregation. The NIR reflectances of ZnO-80/1, ZnO-40/1, and ZnO-20/1 particles were 77%, 57% and 29%, respectively. The ZnO-80/1 particles showed the highest NIR reflectance as their morphology has a lower tendency to aggregate [26]. In addition, as the wavelength of light increased, the reflectance of pigments in the NIR region decreased. The reflection in the NIR region is indicative of the NIR-shielding property of ZnO particles. The ZnO used in the NIR-shielding application should have high NIR reflectance to prevent the passage of NIR rays, while the glass coating application should have a low visible reflection to allow light to pass through in order to increase the brightness inside the building. With this condition, it was found that a combination of ZA and ZN precursors improved the optical properties of the ZnO particles compared to use of either precursor alone, not only resulting in decreased visible reflectance but also increased NIR reflectance.

### 3.4. Thermal Insulation Test

**Figure 8:**
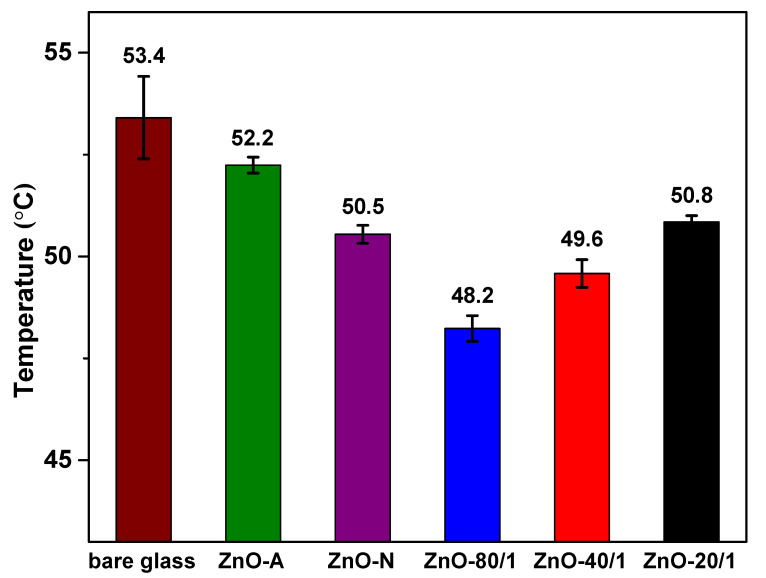
The thermal insulation test of ZnO prepared from different ratios of ZA/ZN precursors. The surrounding temperature was 70 ± 2 °C.

The thermal insulation test was designed to assess the heat prevention performance of the prepared ZnO particles. It consisted of a PS foam box, a sample coated on a glass substrate, and an IR lamp, which mimic a building, window glass, and sunlight, respectively. After shining the IR lamp onto the PS foam box, NIR rays with high thermal energy penetrate through the window glass, causing an increase in temperature inside the building. The coated samples with NIR-shielding properties could lower the inside building temperature. The thermal insulation results of all the ZnO particles are shown in Figure 8. It can be seen that a neat glass slide exhibited the highest inner temperature, while all the ZnO coated on the glass samples presented a comparatively lower temperature. From the setup, ZnO-N was found to result in a significantly lower temperature inside the box than ZnO-A, which is due to the fact that ZnO-N has a larger particle size (in the submicron scale) and, therefore, better NIR reflection performance than ZnO-A. This observation is in good agreement with the UV-Vis-NIR results. Moreover, the inner temperature of the foam box was 48.2 ± 0.3, 49.6 ± 0.3, and 50.8 ± 0.2 °C for the ZnO-80/1, ZnO-40/1, and ZnO-20/1 powders, respectively. According to the results, it was clear that the ZnO-80/1 powder had the lowest inner temperature in the foam box, 5.2 °C lower than that of neat glass, while the ZnO-40/1 and ZnO-20/1 powders had temperatures that were only 3.8 and 2.6 °C lower, respectively. The combination of ZA and ZN precursors improved the NIR barrier performance of ZnO more than compared with the use of pure ZA or ZN precursors. In other words, the mixture of ZA and ZN precursors with extreme mole ratios, such as 80 to 1 or even 20 to 1, showed a synergistic effect that can considerably reduce the inner temperature in glass buildings.

**Figure 9:**
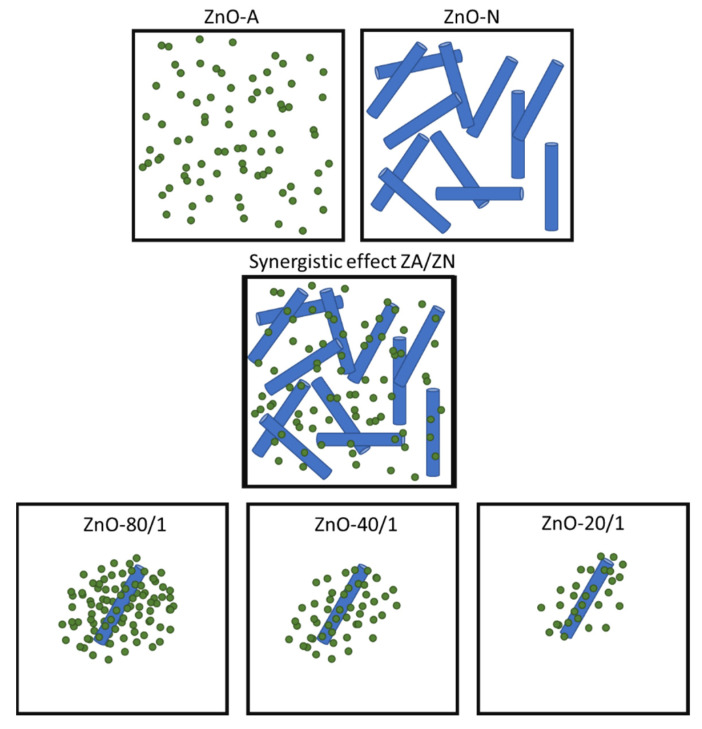
The model proposed for synergistic morphology resulting from the use of mixed zinc precursor in ZnO synthesis. The small particles of ZnO-A from the ZA precursor fill the voids of ZnO-N submicrorods from the ZN precursor.

The synergistic effect is a result of the arrangement between small particles and a very low proportion of submicrorod particles prepared from ZA and ZN precursor solutions. The results from the SEM and thermal insulation tests of all ZnO samples showed that ZnO-A had small particles, which decreased the active site area for reflecting NIR radiation. Although ZnO-N particles had a larger active site area than ZnO-A particles and could therefore lower the inner building temperature more effectively, there were voids between the ZnO submicrorod particles. With the combination of ZA and ZN solutions, the addition of the small particles to the voids of the submicrorod particles significantly increased the active site area for solar reflection in the mixed samples, more so than when using only one zinc precursor. A model for the synergistic morphology of the mixed zinc precursor conditions is proposed, as shown in Figure 9. Interestingly, as the amount of ZA increased, the inner temperature of building decreased, which is due to the fact that more small particles filled the voids between the submicrorods, hindering the passage of NIR radiation through the window glass. The synergistic morphology of the mixed zinc precursors contributed to a higher active site for reducing NIR radiation penetration and thus lowered the buildup of heat that is responsible for increasing the indoor temperature of buildings.

## 4. Conclusions

The preparation of zinc oxide particles from a simple precipitation process yielded small and submicrorod particles using zinc acetate and zinc nitrate precursors, respectively. ZnO-80/1, ZnO-40/1, and ZnO-20/1 produced in this study were characterized by small particles. The average particle sizes of ZnO-80/1, ZnO-40/1, and ZnO-20/1 were 655, 640, and 620 nm, respectively. The optical properties of the ZnO powders were found to be strongly affected by the proportions of the two zinc precursors. The ZnO-80/1 had a compact coral-sheet structure with a large active site area. This unique morphology of the ZnO-80/1 powder was highly capable of reflecting NIR rays, leading to the highest measured NIR reflectance of 77%, while those of ZnO-40/1 and ZnO-20/1 were only 57% and 29%, respectively. Thermal insulation testing of all ZnO powders demonstrated that the inside temperatures of the box were considerably higher for the ZnO powders prepared from the pure zinc precursors (ZnO-A and ZnO-N) than those prepared using a mixture of zinc precursors. In a comparison of the ZnO-40/1 and ZnO-20/1 powders, coating with ZnO-80/1, or with the coral-sheet structure, could reduce the inner temperature of the building more than the aggregated structures of ZnO-40/1 and ZnO-20/1, which decreased the active site area for reflecting NIR radiation. Lastly, the ZnO-80/1 powder could reduce the inner temperature to 48.2 °C, which was 5.2 °C lower than that of the neat glass substrate without any coating. The integrations of low visible and high NIR reflectance features of prepared ZnO powders with various mole ratios of zinc precursors have potential industrial applications in thermal insulation.

## Figures and Tables

**Table 1:** The structure parameter and crystallite size of ZnO powders of (101) plane.

Sample	2Theta Position (Degree)	FWHM (Degree)	Crystallite Size (nm)	d-Spacing (nm)
ZnO-A	36.2446	0.2282	37	0.2477
ZnO-N	36.2402	0.4025	21	0.2477
ZnO-80/1	36.2288	0.2452	34	0.2478
ZnO-40/1	36.2361	0.2652	32	0.2477
ZnO-20/1	36.2332	0.2768	30	0.2477

## Data Availability

Not applicable.
